# Revolutionary Science

**DOI:** 10.1128/mBio.00158-16

**Published:** 2016-03-01

**Authors:** Arturo Casadevall, Ferric C. Fang

**Affiliations:** a Department of Molecular Microbiology and Immunology, Johns Hopkins Bloomberg School of Public Health, Baltimore, Maryland, USA; b Departments of Laboratory Medicine and Microbiology, University of Washington School of Medicine, Seattle, Washington, USA

## Abstract

On rare occasions in the history of science, remarkable discoveries transform human society and forever alter mankind’s view of the world. Examples of such discoveries include the heliocentric theory, Newtonian physics, the germ theory of disease, quantum theory, plate tectonics and the discovery that DNA carries genetic information. The science philosopher Thomas Kuhn famously described science as long periods of normality punctuated by times of crisis, when anomalous observations culminate in revolutionary changes that replace one paradigm with another. This essay examines several transformative discoveries in the light of Kuhn’s formulation. We find that each scientific revolution is unique, with disparate origins that may include puzzle solving, serendipity, inspiration, or a convergence of disparate observations. The causes of revolutionary science are varied and lack an obvious common structure. Moreover, it can be difficult to draw a clear distinction between so-called normal and revolutionary science. Revolutionary discoveries often emerge from basic science and are critically dependent on nonrevolutionary research. Revolutionary discoveries may be conceptual or technological in nature, lead to the creation of new fields, and have a lasting impact on many fields in addition to the field from which they emerge. In contrast to political revolutions, scientific revolutions do not necessarily require the destruction of the previous order. For humanity to continue to benefit from revolutionary discoveries, a broad palette of scientific inquiry with a particular emphasis on basic science should be supported.

## EDITORIAL

Revolution doesn’t have to do with smashing something—it has to do with bringing forth something.

—Joseph Campbell

Any discussion of revolutionary science must begin with Thomas Kuhn, who popularized the notion of paradigm change in his enormously influential treatise *The Structure of Scientific Revolutions*, first published in 1962 and subsequently expanded (1). Kuhn argued that scientific revolutions occur when a crisis in normal science resulting from unresolved anomalies causes a paradigmatic shift in a world view. In developing his arguments, Kuhn drew heavily on examples from the physical sciences, such as the Copernican revolution with its shift from a geocentric to a heliocentric viewpoint. Over the past half-century, some of Kuhn’s concepts have been criticized, including his distinction between normal and revolutionary science (2, 3). One of the greatest problems with the Kuhnian view of revolutionary science is that it does not account for other types of scientific revolution, particularly those in biology (4). Neither the theory of evolution, nor the germ theory of disease, nor the discovery that DNA carries genetic information seems to have been triggered by the type of crisis in normal science that he envisioned. Here we visit the subject of revolutionary science as part of our continuing exploration of the state of current science that has included prior essays on descriptive (5), mechanistic (6), important (7), specialized (8), diseased (9), competitive (10), (a)historical (11), and field (12) science.

## WHAT IS REVOLUTIONARY SCIENCE?

In considering revolutionary science, we begin with the definition of the word. “Revolutionary” is derived from the Latin word *revolutionem*, which referred to a turning motion and was originally used in relation to celestial bodies (13). The *Oxford English Dictionary* gives several definitions of the word, of which the most useful for our purposes is “a dramatic or wide-reaching change” or the “overthrow of an established … order by those previously subject to it.” Kuhn did not provide specific criteria to distinguish revolutionary from normal science and often refers to revolutionary and “extraordinary” science as if they are synonymous; others have similarly discussed revolutionary science without explicitly defining it (2, 14). Charlton proposed that major prizes be used to define and measure revolutionary science (15–17), but we are concerned that not all great discoveries are recognized by awards (18), nor are all award-winning discoveries revolutionary (19).

It is noteworthy that revolutions in politics not only change the political system but also affect other areas of human endeavor. Both the American and French revolutions in the late 18th century and the Russian and Chinese revolutions in the 20th century replaced prior systems of government with new forms and affected other human endeavors, including the relationship between church and state and the social order. Furthermore, each revolution also had major and immediate repercussions for other nations. In science, the Copernican revolution similarly signaled the end of a geocentric view of the world and its replacement with a heliocentric model. The success of the heliocentric model, together with other observations, broke the notion that received wisdom from antiquity was certain and reliable, thus opening the way for additional questioning in other areas of natural philosophy that ultimately ushered in the scientific revolution of the 17th century. Heliocentric theory also affected other important disciplines, such as theology, astronomy, and astrology, and directly impacted the calculation of the calendar.

We propose a definition of revolutionary science as a conceptual or technological breakthrough that allows a dramatic advance in understanding that launches a new field and greatly influences other fields of science. The Darwin-Wallace theory of evolution therefore qualifies as a revolution because it spawned the new field of evolutionary biology and profoundly influenced diverse fields, including anthropology, theology, sociology, and political science, soon after its publication in 1859. The discovery that DNA is the transforming principle of heredity and the subsequent elucidation of its structure also meet our criteria for revolutionary science because they launched the field of molecular biology while transforming the fields of genetics, medicine, and biochemistry. Eventually, this revolutionary discovery would even resonate in the fields of criminology, anthropology, and history, where it was used to solve crimes, clarify relationships between human populations, and determine the paternity of Thomas Jefferson’s descendants. The discovery that DNA is the transforming principle had an immediate impact on numerous fields of scientific inquiry and within a generation had given rise to technologies to produce new pharmacological products such as human insulin.

Revolutionary science is not synonymous with extraordinary or important science (7). For example, the discovery of reverse transcriptase upended the central dogma of molecular biology, explained how RNA viruses replicate, and provided a target for some of our most important antiretroviral drugs. The discovery was recognized with a Nobel Prize. By any measure, this is extraordinary and important science. A yet, in our view, the discovery of reverse transcriptase does not qualify as a scientific revolution, as the discovery occurred within the established field of molecular biology and did not launch a new discipline or significantly affect disciplines outside virology and closely related fields. Similarly, targeted genome editing with CRISPR/Cas9 is a technology of tremendous importance that is rapidly having an impact in many fields associated with molecular biology. However, at this time, this discovery does not yet meet the criteria for revolutionary science, although it remains possible that subsequent events will establish it as a revolutionary finding. In contrast, the invention of the PCR meets our criteria for revolutionary science because it spawned new fields such as forensic DNA analysis and provided a transformative tool for such unrelated fields as anthropology, archeology, criminology, and historical analysis. We note that other accepted revolutions in science also appear to meet our criteria (Table 1).

**TABLE 1:** Characteristics and impact of scientific revolutions

Revolution	Yr	Nobel Prize^a^	Type	New field(s)	Affected field(s)	Time (yr) to impact^b^
Heliocentric solar system	1543	NA	Conceptual	Astronomy	Theology	~100
Light microscopy	1600s	NA	Experimental	Microbiology, cytology	Biology, anatomy, physiology	70
Newtonian mechanics	1687	NA	Conceptual	Classical mechanics, calculus	Physics, astronomy, mathematics	10–20
Vaccination	1796	NA	Experimental	Vaccinology	Medicine, public health	Variable
Computers	1822	NA	Experimental	Computer science	All fields	>100
Thermodynamics	1824	NA	Experimental	Classical thermodynamics	Chemistry, physics, engineering, geology	30
Electromagnetism	1820	NA	Experimental	Electrodynamics	Physics, engineering	10
Natural selection	1859	NA	Conceptual	Evolution	Biology, political science, theology	10
Germ theory	1850s–1870s	NA	Experimental	Infectious diseases, epidemiology	Public health, immunology	20–30
Mendelian inheritance	1866	NA	Conceptual-experimental	Genetics	Biology, botany, medicine	35
Phagocytosis, antibodies	1882–1890	Y	Experimental	Immunology	Medicine	5–10
Filterable viruses	1890s	Y	Experimental	Virology	Microbiology, medicine, public health	5
X-rays	1895	Y	Experimental	Radiology, X-ray spectroscopy, X-ray crystallography	Astronomy, medicine, dentistry	15–20
Radioactivity	1896	Y	Experimental	Radiation biology, radiometric dating, nuclear medicine, nuclear engineering	Anthropology, archaeology, history, military science, medicine	10
Quantum theory	~1900	Y	Conceptual- experimental	Quantum mechanics, quantum chemistry, quantum information	Classical physics, chemistry, electronics, biology	10
Relativity	1905–1920	Y	Conceptual	Relativity	Atomic physics, nuclear physics, quantum mechanics, astronomy, cosmology	10–20
Continental drift	1912–1970	N	Conceptual	Plate tectonics	Geology, evolutionary biology	10
Laser physics	1917–1960	Y	Conceptual- experimental	Nonlinear optics	Astronomy, biology, chemistry, medicine, physics	5
Transistor	1947	Y	Experimental	Solid-state electronics	Computer science	5–10
Heredity from DNA	1944–1953	Y	Experimental	Molecular biology	Genetics, medicine, biochemistry	10
Prions	1960s–1980s	Y	Experimental	Prion biology	Biochemistry, microbiology, neurology, veterinary medicine	20–30
DNA sequencing	1970	Y	Experimental	Genomics	Biology, medicine, forensics	5–10
Molecular cloning	1972	Y	Experimental	Recombinant DNA	Biology, medicine	5
Three domains of life	1977	N	Experimental	Archaeal biology, molecular taxonomy	Microbial ecology, evolutionary biology	10
PCR	1987	Y	Experimental	Molecular forensics, molecular diagnostics, synthetic biology	Molecular biology, medicine, anthropology, archeology, forensics, history	5

a NA, not applicable; Y, yes; N, no.

b Estimates based on history of the field and the historical record.

Some of the scientific revolutions listed, like the Copernican, Newtonian, Einsteinian, and molecular biology revolutions, are already well established in the pantheon of revolutionary science. Others, such as plate tectonics and the development of the PCR, may not quite be chiseled into the pantheon walls but clearly meet our criteria. We acknowledge that such a list is somewhat subjective and some inclusions or omissions can be debated. One area of potential controversy is whether a revolutionary technology qualifies as revolutionary science. For example, the invention of the transistor might be considered a technological innovation rather than a scientific revolution. The discovery was catalyzed by an industrial effort to improve upon vacuum tube technology at Bell Laboratories. However, the transistor led to the new scientific discipline of solid state physics, and its importance for modern electronics had an enormous impact on many aspects of society, which argues for its inclusion as revolutionary science. The microscope, the telescope, the laser, and the PCR are other examples of technologies that have allowed scientists to ask new questions that have transformed our understanding of the world. The interested reader is welcome to add to or subtract from our list.

Our definition provides a straightforward means to demarcate revolutionary and nonrevolutionary science. However, it is interesting to note that the pace of scientific revolutions can vary widely. The Copernican revolution took almost a century to unfold, whereas the molecular biology revolution occurred within a decade and the PCR revolution began to influence criminology within a few of years of publication. Mendel’s findings, despite their fundamental importance to genetics, were extremely delayed in their influence, as illustrated by the fewer than five times that his work was cited in the 19th century. The dissemination of scientific information has accelerated markedly since Mendel’s time, but revolutions may still be delayed if there are no adequate experimental tools to test the predictions of a novel theory. Alfred Wegener’s theory of continental drift failed to spark a revolution when proposed in 1912 because he lacked an explanatory mechanism; the plate tectonics revolution only became possible half a century later, when technological advances allowed the demonstration of sea floor spreading. Nevertheless, the theory of plate tectonics is truly revolutionary science, as it created a new field with tremendous explanatory power that has also profoundly influenced paleontology, evolutionary biology, oceanography, and even astronomy, with regard to our understanding of crustal dynamics on other moons and planets.

Confusion may occur when words have different meanings in common parlance and in science, such as the words “chaos,” “error,” and “significant,” and here we note the limitations of “revolution” as a metaphor when used to describe a transformative scientific discovery. As Stephen Jay Gould memorably observed, “Great revolutions smash pedestals” (20), and political revolutions destroy one social order to allow its replacement with another. Scientific revolutions, in contrast, do not necessarily destroy or invalidate earlier work but rather place it in a new light. Old observations can be newly understood in the context of a novel paradigm. Einstein’s theory of relativity did not destroy Newtonian mechanics but rather demonstrated their limitations. Newtonian mechanics were still used to get a man to the moon. Moreover, although some have interpreted Kuhn’s analogy of revolution to indicate that science is merely a social construct that does not make cumulative progress (21), science exhibits a strong tendency to build upon, not to discard, what has come before, particularly as fields mature and coalesce around a consensus paradigm.

## WHAT MAKES REVOLUTIONARY SCIENCE DIFFERENT FROM NORMAL SCIENCE?

Kuhn posited that extraordinary science, which could lead to revolutionary science, differs fundamentally from normal science, and this distinction has taken hold in the zeitgeist, as evidenced by its frequent mention by essayists. “Normal science” was characterized as routine day-to-day research focused on what Kuhn called “puzzle solving.” Although he later denied any feelings of condescension (22), Kuhn also compared scientists engaged in normal work to “the typical character of Orwell’s *1984*” (1). Karl Popper, in many respects Kuhn’s adversary, was even more dismissive of normal science, describing it as “the activity of … the not-too-critical professional: of the science student who accepts the ruling dogma of the day; who does not wish to challenge it; and who accepts a new revolutionary theory only if almost everybody else is ready to accept it—if it becomes fashionable by a kind of a bandwagon effect” (23). Kuhn’s depiction of normal science was controversial even in its time (2, 3). His and Popper’s descriptions appear to be caricatures of science, if, in fact, they describe science at all. While scientists certainly do attempt to solve problems, they are constantly testing existing dogmas and, indeed, hoping to find evidence that current thinking may need to be revised, even if the revisions are more modest than a full-fledged scientific revolution.

The way in which we have defined revolutionary science provides a new perspective from which to view Kuhn’s claim. First, revolutionary science cannot be identified at the moment of discovery since the implications and consequences of a finding are only evident after the passage of time. Second, if revolutionary science cannot be distinguished from nonrevolutionary science at the moment of discovery, this implies that there is no fundamental qualitative or quantitative difference between the two. By way of illustration, let us examine the discovery of the structure of DNA and the scientists working on the problem using similar techniques, including Watson, Crick, Franklin, Wilkins, and Pauling. These researchers were attempting to incorporate Chargaff’s observations regarding the relative amounts of purine and pyrimidine bases into a chemical structure for DNA, which is, in essence, a puzzle that fits within Kuhn’s view of normal science. The technological breakthrough of X-ray fiber diffraction allowed investigators to produce structural models that integrated the X-ray data with biochemical constraints such as Chargaff’s ratios and the acidic pH of DNA. Although Pauling published first, his model of a three-stranded structure was not consistent with chemical observations and was rapidly discarded. Rosalind Franklin obtained the best diffraction data, which were used by Watson and Crick to propose their double-helix model. Watson and Crick’s efforts involved false starts and a remarkable piece of luck—Watson shared an office with Jerry Donahue, a chemist who noticed that he was using the wrong tautomeric structures for the bases and provided a key insight by providing base structures that allowed complementary pairing. Watson and Crick also benefited when Wilkins shared Franklin’s unpublished diffraction data with them. It is difficult to view the sinuous trail of discovery punctuated by false starts and serendipity and regard this as epistemically distinctive from what was being done in other laboratories. Had Watson and Crick not proposed their double-helix model, it is virtually certain that another group would have eventually stumbled onto the correct model of DNA. Watson and Crick were honored for their discovery with a Nobel Prize in 1962, the same year when Kuhn’s work was published. This biological revolution resulted from puzzle solving, or “normal science,” without any paradigmatic crisis. Hence, the Kuhnian notion of a separation between normal and revolutionary science does not apply to what is perhaps the most important biological finding of the 20th century.

## THE TRUE NATURE OF SCIENTIFIC REVOLUTIONS

A survey of scientific revolutions (Table 1) suggests that scientific revolutions have developed in a variety of ways. Some, indeed, seem to correspond to Kuhn’s description, such as the discoveries of filterable viruses and prions, in which the progressive accumulation of anomalous observations led to a crisis that culminated in the generation of a new paradigm. However, many others do not correspond to such a scenario. The theory of evolution was an intellectual synthesis suggesting an explanation for biological variation, which made its debut without a mechanism. In contrast to Wegener’s theory of continental drift, which initially failed to gain traction because no one could imagine how continents could move, the Darwin-Wallace theory of evolution captured the public imagination and gradually gained acceptance among scientists despite the initial absence of a mechanism. The germ theory of disease began with speculation and eventually emerged in mature form from the contributions of researchers in multiple countries who were investigating such disparate phenomena as silkworm disease, cholera, childbed fever, and ringworm. Such observations were eventually able to conclusively link specific diseases to certain microbes. Acceptance of the germ theory required decades of work involving both observation and experimentation, as exemplified by John Snow’s investigation of a London cholera outbreak and the transmission of anthrax by Koch. In contrast to continental drift, the germ theory of disease was accepted despite the lack of a mechanism to explain why some microbes could be pathogenic to some individuals yet harmless to others, a problem that continues to vex the field of microbial pathogenesis. The molecular biology revolution required both intellectual and experimental advances that culminated in the identification of DNA as the agent of heredity and the determination of its structure, which in turn provided a mechanism to explain the transfer of information. The invention of the transistor at Bell Laboratories arose from experimental observations and transformed the field of electronics. The PCR revolution was a technological innovation that allowed the amplification of small segments of DNA, which was enabled by the availability of a thermostable polymerase. Although Kary Mullis was recognized for the discovery of PCR (24), it is noteworthy that the concept of denaturing and replicating DNA with synthetic primers had been published over a decade earlier (25), and the concept could not have been successfully realized without the preceding isolation of a thermophilic microorganism with a thermostable DNA polymerase (26). Hence, PCR emerged from normal science using established facts that were assembled into an extraordinary idea, which ushered in a revolution. Kary Mullis has stated that his inspiration came during a night drive on California State Highway 128, when the air was redolent with flowering buckeye (27). Although other aspects of PCR required careful attention to detail to become facile and useful, the PCR revolution appears to have begun by inspiration.

The most striking aspect of the revolutions in Table 1 is the absence of any common structure to explain their occurrence or to define their nature. Revolutionary science can emerge from careful observation and description, experimentation, thought, or inspiration and often requires a combination of these elements, seasoned with a touch of serendipity. The only common denominator of all scientific revolutions is that they resulted from human curiosity and an unceasing drive to understand the natural world.

## SOCIETY AND REVOLUTIONARY SCIENCE

Scientific revolutions have had tremendous practical benefits for society (Table 2). The human population has increased exponentially since the mid-18th century, around the time when scientific inquiry became firmly established as a foundation for many human activities. Humanity has repeatedly avoided a Malthusian crisis by increasing the efficiency of food production, a direct result of the industrial revolution coupled with advancements in farming, crop varieties, and food preservation, which themselves have benefited from other revolutions. For example, the ability to preserve food by canning was made possible by the industrial and germ theory revolutions, which in turn allowed food to be consumed long after it was produced and stored in sealed containers that were free of botulism. Since the late 19th century, the pursuit of science began to be supported by public funds, first in Germany and then in numerous other countries, including the United States, particularly during and after World War II. We note that more than half of the scientific revolutions listed in Table 1 occurred with public support. Furthermore, the linkage between revolutionary scientific findings and the emergence of measurable public goods (Table 2) provides a direct refutation of the recently expressed viewpoint that public spending on basic science is not associated with technological advances that benefit humankind (28).

**TABLE 2:** Some practical societal benefits from scientific revolutions

Revolution	Societal benefit(s)^a^
Heliocentric solar system	More accurate calendars
Newtonian mechanics, thermodynamics	Industrial revolution, mechanical transportation
Vaccines, phagocytosis, antibodies	Vaccines, passive antibody therapies
Computer	Computers
Natural selection	Comparative anatomy
Light microscopy, germ theory, viruses	Antibiotics, epidemic prevention
X-rays	Diagnostic tests
Radioactivity	Cancer treatment, energy, weaponry, radioactive dating
Quantum theory	Improved electronics
Relativity	Global positioning system
Continental drift	Seismological forecasting
Laser physics	Medical applications, printing, information management, communications
Transistors	Solid-state electronics
Heredity from DNA, molecular cloning, DNA sequencing	New therapeutic agents, genetically modified plants and animals, molecular diagnosis of birth defects
PCR	Biotechnology, forensic analysis, diagnostic tests

a Not a complete list.

## FOSTERING REVOLUTIONARY SCIENCE

As society is both the beneficiary and major sponsor of revolutionary science (Table 2), it is important to consider how revolutionary science can best be encouraged. Are there lessons from earlier scientific revolutions that can hasten the pace of scientific discovery? Although 25 revolutions are too small a sample from which to draw firm conclusions, some themes are discernible. First, although scientific revolutions are often associated with individual scientists, a closer inspection reveals that each of these individuals required a community of scientists making observations and raising questions that contributed to a revolutionary discovery. Second, a striking interdependence of scientific disciplines is evident in the genesis of certain scientific revolutions. For example, the molecular biology revolution depended upon advances in physics applied to molecular structures (X-ray diffraction), microbiology (pneumococcal transformation), chemistry (bases, amino acids, pH), biochemistry (Chargaff rules), mathematics (fiber diffraction analysis), and a well-supported academic system to provide scientists with sufficient time and resources to pursue their curiosity. Third, scientific revolutions are reliant on both routine scientific pursuits and moments of brilliant insight. The theory of natural selection was critically dependent on the assembly of a large amount of descriptive observations on species variation obtained through the mundane actions of specimen collection, characterization, classification, and archiving, activities that no biological scientist would consider extraordinary. However, when the information gathered from these mundane activities is illuminated and unified by an extraordinary thought, a larger synthesis emerges that constitutes revolutionary science. Similarly, neither the isolation of thermophiles from hot springs not the report that their enzymes are thermostable might seem, on the surface, to be extraordinary, yet without these findings, the PCR revolution could not have taken place. Fourth, the majority of the scientific revolutions listed in Table 1 emerged from inquiries into problems of basic science. This suggests that society must support research in broad fields of inquiry, with a major emphasis on basic science, in order to create the fertile substrate from which tomorrow’s scientific revolutions will arise.

In closing, we emphasize that this essay is not intended as a rejection of the seminal contributions of Thomas Kuhn. Although our definition of revolutionary science implies that there is no essential distinction between revolutionary and normal science and our argument that scientific revolutions lack a common structure may be seen to challenge the conclusions of *The Structure of Scientific Revolutions* (1), we have had the benefit of an additional half-century of scientific history for analysis, including four biological revolutions, as well as access to many thoughtful discussions and criticisms of Kuhn’s views. This essay is made possible by the intellectual spaces that Kuhn created. In a sense, we are following Kuhn’s directive by challenging his paradigm, as we have found it to be insufficient to explain the multifarious nature of scientific revolutions. The philosophy of science, like science itself, is a work in progress, and the analysis of the nature of science can be anticipated to evolve with additional human experience. We encourage a continuing dialogue among historians, philosophers sociologists, economists, and working scientists in an ongoing effort to understand this essential human institution that constitutes science and to foster an environment conducive to revolutionary science.
